# A serum proteomic study of two case-control cohorts identifies novel biomarkers for bipolar disorder

**DOI:** 10.1038/s41398-022-01819-y

**Published:** 2022-02-08

**Authors:** Andreas Göteson, Anniella Isgren, Timea Sparding, Jessica Holmén-Larsson, Joel Jakobsson, Erik Pålsson, Mikael Landén

**Affiliations:** 1 Department of Psychiatry and Neurochemistry, Institute of Neuroscience and Physiology, University of Gothenburg, Gothenburg, Sweden; 2 Department of Medical Epidemiology and Biostatistics, Karolinska Institutet, Stockholm, Sweden

**Keywords:** Diagnostic markers, Bipolar disorder

## Abstract

We set out to identify novel protein associations with potential as clinically viable biomarkers for bipolar disorder. To this end, we used proximity extension assay to analyze 201 unique proteins in blood serum from two independent cohorts comprising patients with bipolar disorder and healthy controls (total *n* = 493). We identified 32 proteins significantly associated with bipolar disorder in both case-control cohorts after adjusting for relevant covariates. Twenty-two findings are novel to bipolar disorder, but 10 proteins have previously been associated with bipolar disorder: chitinase-3-like protein 1, C-C motif chemokine 3 (CCL3), CCL4, CCL20, CCL25, interleukin 10, growth/differentiation factor-15, matrilysin (MMP-7), pro-adrenomedullin, and TNF-R1. Next, we estimated the variance in serum protein concentrations explained by psychiatric drugs and found that some case-control associations may have been driven by psychiatric drugs. The highest variance explained was observed between lithium use and MMP-7, and in post-hoc analyses and found that the serum concentration of MMP-7 was positively associated with serum lithium concentration, duration of lithium therapy, and inversely associated with estimated glomerular filtration rate in an interaction with lithium. This is noteworthy given that MMP-7 has been suggested as a mediator of renal tubulointerstitial fibrosis, which is characteristic of lithium-induced nephropathy. Finally, we used machine learning to evaluate the classification performance of the studied biomarkers but the average performance in unseen data was fair to moderate (area under the receiver operating curve = 0.72). Taken together, our serum biomarker findings provide novel insight to the etiopathology of bipolar disorder, and we present a suggestive biomarker for lithium-induced nephropathy.

## Background

Bipolar disorder is a prevalent (~2.4% [1]) affective syndrome with severe impact on life quality [2] and mental as well as physical health [1]. Clinical decision-making and diagnostics rely on clinical presentation and structured interviews with no objective measures (e.g., protein biomarkers). Although bipolar disorder type 1 (patients with at least one manic episode) is one of the more reliable diagnoses in psychiatry [3], early stages of the disorder and other bipolar subtypes present diagnostic challenges. Moreover, although commonly prescribed pharmaceutics are generally safe and effective, some drugs have feared adverse effects where few predictive biomarkers exist [4]. A notable example is the renal side-effects of lithium [5]. Objective measures are hence needed for early and reliable diagnoses of bipolar disorder and for monitoring adverse drug effects.

The etiopathology of bipolar disorder is largely unknown. Recent progress in psychiatric genomics has demonstrated shared liability across psychiatric disorders [6], which extends to biomarker studies where many findings are shared across disorders [7–9]. Previous blood-based proteomic studies have identified aberrations in several inflammatory markers, neurotrophins, and oxidative stress markers [10]. Some studies have developed composite biomarker panels aiming at distinguishing between psychiatric disorders [7, 11–15] or classifying mood states [16, 17]. One study derived a promising biomarker panel for the classification of both pre-diagnostic and misdiagnosed cases with bipolar disorder [18] but no diagnostic biomarkers have yet been validated for clinical use.

The aim of this study was to identify novel biomarker candidates for bipolar disorder. To this end, we analyzed blood serum samples from two independent case-control cohorts of bipolar disorder (total *n* = 493), employing a multiplexed immunoassay-based approach targeted towards proteins involved in a broad set of disease processes (201 proteins in total).

## Methods

### Study cohorts and ethics

The St. Göran bipolar project (SBP) is a multi-disciplinary longitudinal observational study of persons with bipolar syndromes and healthy controls, as previously described [19–22]. The study comprises two independent cohorts where patients are enrolled at bipolar outpatient units in Stockholm (SBP-S) and Gothenburg (SBP-G). All patients were assessed using the standardized interview protocol Affective disorders evaluation, which was developed for the Systematic Treatment Enhancement Program of Bipolar Disorder (STEP-BD) study [23]. The Affective disorders evaluation guides the interviewer through a systematic assessment of the patient’s current mental state, past history, and diagnostic criteria according to DSM-IV as contained in the Structured Clinical Interview for DSM-IV-Axis I Disorders (SCID-I). Co-morbid psychiatric disorders were screened for using the Mini-International Neuropsychiatric Interview (M.I.N.I.). The full diagnostic assessment was based on all available sources of information, including patient interview, case records, and interviews with the next of kin when possible. A final best estimate diagnostic decision was made by board-certified psychiatrists specialized in bipolar disorder. The lifetime severity of bipolar disorder was rated at the interview using the Clinical Global Impression (CGI) rating scale. The Montgomery-Åsberg Depression Rating Scale (MADRS) [24] and the Young mania rating scale (YMRS) [25] were used to gauge mood symptoms at blood sampling date. For ethical reasons in this non-interventional study, patients continued to take their prescribed medications.

The inclusion criteria were age ≥18 years and a DSM-IV bipolar spectrum diagnosis (type 1, type 2, not otherwise specified, cyclothymia, or schizoaffective syndrome bipolar form), whereas inability to complete the study protocol or provide informed consent rendered exclusion.

Control participants were recruited by Statistics Sweden (www.scb.se) by sending an invitation letter to randomly selected individuals from the general population living in the same catchment area as enrolled patients. Responders were first screened for exclusion criteria in a telephone interview. Eligible persons were scheduled for a visit and further interviewed by an M.D. using the M.I.N.I. and selected parts of the Affective Disorder Evaluation. Exclusion criteria for control participants were any current psychiatric disorder or use of psychiatric medications, bipolar disorder or schizophrenia in first-degree relatives, substance abuse, neurological conditions except mild migraines, untreated endocrine disorders, and pregnancy. Controls with past history of an isolated depressive episode, isolated episode of panic disorder, as well as a mild eating disorder or obsessive-compulsive disorder that remitted spontaneously or with brief psychotherapeutic counseling were not excluded.

The SBP-study has been approved by the Regional Ethics Committee in Stockholm and all study participants provided oral and written informed consent to participate.

### Blood sampling and proximity extension assay analysis

Blood samples were collected in fasting subjects between 8–9 AM and were allowed to clot in room temperature for 30–60 min pending centrifugation (10 min at 1700 × *g*). In SBP-G, the supernatant was immediately stored in a local −70 °C freezer awaiting bulk transport to the biobank. In SBP-S, the supernatant was kept in low temperature (<5 °C) pending direct transport to the biobank within 4 h for long-term storage at −70 °C.

Blood serum samples from 342 individuals in SBP-S and 157 individuals in SBP-G were analyzed by Olink^®^ Proteomics using Proseek 96-plex protein panel kits for biomarker discovery, covering a total of 201 unique proteins. This technique builds on proximity extension assay, where paired oligonucleotide-labeled antibody binding is coupled with a quantitative polymerase chain reaction (qPCR) readout, enabling a multiplex setup with high specificity and quantitative accuracy [26]. Here, we used a broad set of protein panels covering disease processes in cardiovascular disease (CVD1, v.2002), inflammation (INF, v.3001), and oncology (ONCIv2, v.4001).

The two cohorts were analyzed and processed separately over several plates (4 in SBP-S, 2 in SBP-G) with cases and controls randomized across plates. Laboratory technicians were blinded to clinical data. Internal plate standardization and quality control were performed by Olink (https://www.olink.com/resources-support/white-papers-from-olink/), exporting normalized protein expression (NPX) values on log2-scale [26]; a one unit increase in NPX corresponds to a two-fold increase in analyte concentration. Due to technical issues, three samples from one plate and the assay brain-derived neurotrophic factor (BDNF) failed in the experimental protocol and were excluded by Olink. To further account for batch effects, the NPX-values were median centered per assay and plate. There was a strong correlation across panels for the 65 protein assays that overlapped across panels (median *r* = 0.91 and 0.97 in SBP-S and SBP-G, respectively). NPX-values from the panel with the least amount of quality control flags were kept for each overlapping assay. Four additional samples from one plate each were excluded due to clear and consistent deviation of values from one panel compared to the other panels in overlapping assays, or due to being labeled “warning” in the Olink quality control together with an outlier score in the principal component analysis (PCA). Values from overlapping assays were imputed where applicable. No protein concentrations were significantly (*P* < 0.05) correlated with blood sampling date in both cases and controls in any of the cohorts. Protein assays with >20% of values below the limit of detection in both cases and controls were excluded, rendering a final set of 178 proteins in 338 individuals in SBP-S, and 171 proteins in 155 individuals in SBP-G. Supplementary Table 1 lists all studied proteins.

Concentrations of plasma creatinine and serum lithium (S-lithium) were analyzed at the clinical laboratories of Capio (Stockholm, Sweden) and the Sahlgrenska University Hospital (Gothenburg, Sweden) for SBP-S and SBP-G, respectively. The estimated glomerular filtration rate (eGFR) was calculated according to the revised Lund-Malmö study equation with estimated lean body mass [27].

### Statistics

Case-control differences in serum protein concentrations were tested by two-sided *t*-tests adjusting *P*-values for false discovery rate (FDR) [28]. In covariate-adjusted logistic regression models, age, sex, body mass index (BMI), and nicotine usage were included as covariates. Fold change was defined as the mean case-control difference in NPX-scores (log2-scale); a positive value indicates higher concentration in cases than controls and vice versa.

To estimate the influence of psychiatric drugs (see definition in Supplementary material) on our case-control findings, we conducted analysis of variance (ANOVA) models including all four binary drug categories as explanatory variables. *P*-values were Bonferroni-adjusted to conservatively minimize the risk for false positives in this secondary analysis, and eta-squared (*η*^2^) statistics were derived to estimate the proportion of variance in protein concentration explained by each drug category. Post-hoc association analyses with S-lithium concentration, duration of lithium treatment, and eGFR were tested by linear regression adjusted for age and sex. The eGFR model also included case-control status and an interaction term with the use of lithium.

Next, we used a machine learning pipeline to assess the diagnostic potential of a combined set of biomarkers for the classification of the bipolar subtypes and controls. In this analysis, we harmonized and combined data from both cohorts, and excluded bipolar spectrum diagnoses other than type 1 or type 2 (*n* = 30/16 in SBP-S and SBP-G, respectively). We opted for a random forest classification model with 500 trees and tuned hyperparameters (min_n, mtry) to a grid [29]. Model training and evaluation were done in a nested cross-validation design, comprising an inner (25 bootstrap resamples) and an outer loop (fivefold cross-validation with 5 repeats). This procedure keeps control of data leakage by separating tuning, training, and evaluation in the inner and outer loops. The final estimates (*n* = 25, 5 folds × 5 repeats) of classification performance are from unseen test data in the outer loops. We report classification accuracy, area under receiver operating curve (AUROC) [30], Cohen’s kappa, Matthews correlation coefficient (MCC), and the sum of the log loss for each class prediction. We also report the most influential proteins (VIP) in the outer loops according to permutation-based estimations [31].

All analyses were conducted using R (v. 4.0.3) with external packages: arsenal (v. 3.5.0), tidyverse (v. 1.3.0), sjstats (v. 0.18.0), ggplot2 (v. 3.3.2), tidymodels (v. 0.1.2), ranger, (v. 0.12.1), and vip (v. 0.3.2). Code is available at github.com/andreasgoteson.

## Results

### Demographics and clinical characteristics

Two independent case-control cohorts were analyzed in this study, comprising a total of 338 individuals in SBP-S (224 cases and 114 controls) and 155 individuals in SBP-G (100 cases and 55 controls). BMI and nicotine use were significantly higher in cases than controls across both cohorts, whereas no case-control differences were seen for sex or age in any cohort (Table 1). Somatic comorbidities (e.g., asthma, autoimmune disorders, diabetes, hypertonia, hypothyroidism) were more common in cases than controls in both cohorts (*n* = 36:9 in SBP-S and 10:1 in SBP-G in cases and controls, respectively). With respect to subtypes of bipolar disorder, type 1 was more common in SBP-S while type 2 was more common in SBP-G. Further, bipolar disorder participants in SBP-S had more recorded lifetime total mood episodes and longer illness duration but lower illness burden (CGI-S) at interview than cases in SBP-G. A history of psychosis was more common in SBP-S (50%) than in SBP-G (24%), while both antipsychotic and anticonvulsant mood stabilizer usage was more common in SBP-G.

**Table 1:** Demographic and clinical characteristics.

Demographics	SBP-S			SBP-G		
	Bipolar disorder	Controls	*P*	Bipolar disorder	Controls	*P*
*N*	224	114	–	100	55	–
Sex, *n* (%) males^a^	88 (39%)	52 (46%)	0.26	37 (37%)	25 (45%)	0.30
Age, median (IQR)^b^	36.5 (29.0, 49.0)	35.0 (28.0, 44.0)	0.25	39.5 (30.0, 49.0)	45.0 (32.0, 52.0)	0.12
BMI, median (IQR)^b^, *n* = 3 NA	24.9 (22.5, 27.7)	23.3 (21.7, 25.3)	<0.001	26.3 (23.7, 29.8)	25.0 (22.2, 26.7)	0.006
Nicotine use, *n* (%)^a^, *n* = 31 NA	93 (47%)	27 (24%)	<0.001	43 (45%)	14 (25%)	0.018

Clinical characteristics (cases only)	SBP-S	SBP-G
Bipolar subtype, *n* (%)		
Type 1	113 (50%)	36 (36%)
Type 2	81 (36%)	48 (48%)
Bipolar spectrum (other than type 1 or type 2)	30 (13%)	16 (16%)
Total lifetime mood episodes, median (IQR)	11 (6, 23)	27 (13, 48)
Lifetime (hypo)manic episodes, median (IQR)	4 (2, 9)	10 (4, 20)
Lifetime mixed episodes, median (IQR)	0 (0, 0)	0 (0, 10)
Lifetime depressive episodes, median (IQR)	5 (3, 10)	10 (5, 20)
History of psychosis, *n* (%)	105 (50%)	23 (24%)
Duration of illness, median years (IQR)	16 (9, 24)	20 (13, 31)
CGI lifetime, median (IQR)	4 (4, 5)	4 (4, 5)
MADRS, median (IQR)	4 (0, 11)	3 (1, 9)
YMRS, median (IQR)	0 (0, 2)	1 (0, 2)
Comorbid substance abuse, *n* (%)	28 (13%)	4 (4%)
Psychotropic medication		
Lithium, *n* (%)	130 (58%)	49 (49%)
Antipsychotics, *n* (%)	47 (21%)	34 (34%)
Anticonvulsants, *n* (%)	79 (35%)	49 (49%)
Antidepressants, *n* (%)	94 (42%)	53 (53%)

*SBP-S/G* St. Göran bipolar project Stockholm/Gothenburg, *IQR* interquartile range, *BMI* body mass index, *CGI* Clinical Global Impressions Scale, *MADRS* Montgomery-Åsberg Depression Rating Scale, *YMRS* Young Mania Rating Scale, *NA* data not available.

^a^Pearson’s Chi-squared test.

^b^Kruskal–Wallis rank sum test.

### Case-control analyses

**Fig. 1:**
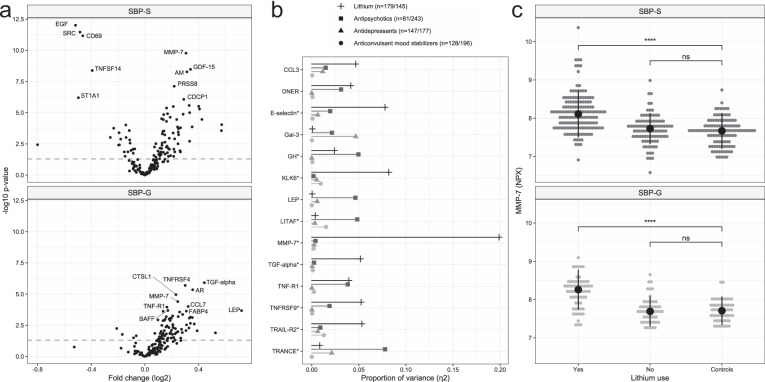
Overview of case-control results and associations with psychiatric drugs. **a** Volcano plots summarizing results from case-control analyses (*T*-test) in both cohorts. The horizontal line indicates *P* = 0.05. The top 10 altered proteins in each cohort are labeled. **b** Proportion of variance (η^2^ from ANOVA models) in protein normalized protein expression (NPX) values explained by each drug category where number of individuals with and without the drug are stated (e.g., 179 individuals were treated with lithium, 145 were not). Proteins with a Bonferroni-adjusted *P*-value < 0.2 are shown. *Bonferroni-adjusted *P* < 0.05. **c** Dot plot showing NPX-values of MMP-7 for each individual stratified by lithium use. *P*-values from *T*-tests. ****P < 0.0001, ns not significant. AM pro-adrenomedullin, AR amphiregulin, BAFF tumor necrosis factor ligand superfamily member 13B, CCL3 C-C motif chemokine 3, CCL7 C-C motif chemokine 7, CD69 early activation antigen CD69, CDCP1 CUB domain-containing protein 1, CTSL1 procathepsin L, DNER Delta and Notch-like epidermal growth factor-related receptor, EGF pro-epidermal growth factor, FABP4 fatty acid-binding protein, adipocyte, Gal-3 Galectin-3, GDF-15 growth/differentiation factor 15, GH somatotropin, KLK6 kallikrein-6, LEP Leptin, LITAF lipopolysaccharide-induced tumor necrosis factor-alpha factor, MMP-7 matrilysin, PRSS8 prostasin, SRC proto-oncogene tyrosine-protein kinase Src, ST1A1 sulfotransferase 1A1, TGF*-*alpha protransforming growth factor alpha, TNFRSF9 tumor necrosis factor receptor superfamily member 9, TNF-R1 tumor necrosis factor receptor superfamily member 1A, TNFRSF4 tumor necrosis factor receptor superfamily member 4, TNFSF14 tumor necrosis factor ligand superfamily member 14, TRAIL-R2 tumor necrosis factor receptor superfamily member 10B, TRANCE tumor necrosis factor ligand superfamily member 11, SBP-S/G St. Göran bipolar project Stockholm/Gothenburg.

In a primary univariate analysis, we tested all studied proteins for case-control differences. A total of 84 proteins in SBP-S and 60 in SBP-G were significantly (FDR < 0.05) associated with bipolar disorder (Fig. 1a, complete results in Supplementary Table 2). Based on these analyses, we included the proteins that passed an exploratory significance threshold (FDR < 0.2) with the equal direction of fold change in both cohorts (*n* = 56) in a logistic regression analysis adjusting results for age, sex, BMI, and nicotine use. Here, 32 proteins differed between cases and controls (*P* < 0.05) in both cohorts and were thus considered replicated (Table 2). These were (in alphabetical order): amphiregulin (AR), C-C motif chemokine 3 (CCL3), CCL4, CCL20, CCL25, chitinase-3-like protein 1 (CHI3L1), C-X-C motif chemokine 16 (CXCL16), CUB domain-containing protein 1 (CDCP1), Fms-related tyrosine kinase 3 ligand (Flt3L), folate receptor alpha (FR-alpha), galectin-3 (Gal-3), growth/differentiation factor 15 (GDF-15), interleukin 10 (IL-10), IL-10 receptor beta (IL-10RB), IL-12, IL-12B, IL-17RB, kallikrein-6 (KLK6), matrilysin (MMP-7), placenta growth factor (PGF), pro-adrenomedullin (AM), procathepsin L (CTSL1), prostasin (PRSS8), protransforming growth factor alpha (TGF-alpha), renin (REN), tumor necrosis factor receptor superfamily member 6 (FAS), tumor necrosis factor ligand superfamily member 13B (BAFF), TNF receptor superfamily member 1A (TNF-R1), TNF-R2, TNFRSF9, TNF receptor superfamily member 10B (TRAIL-R2), and WAP four-disulfide core domain protein 2 (HE4). IL-17RB was the only replicated protein with a lower concentration in cases than controls. A graphical network of protein-protein interactions predicted from public data (www.string-db.org) is shown in Supplementary Fig. 1.

**Table 2:** Case-control analyses. Showing the 32 proteins where serum concentrations were significantly altered between cases and controls in both *T*-test and covariate-adjusted logistic regression, and with equal direction of fold change across the cohorts.

	*SBP-S*					*SBP-G*				
Protein	Fold change	FDR^a^	OR^b^	(95% CI)^b^	*P*^b^	Fold change	FDR^a^	OR^b^	(95% CI)^2^	*P*^b^
Amphiregulin (AR)	0.15	4.5e−02	1.69	1.04–2.82	4.0e−02	0.36	2.5e−04	6.13	2.40–18.03	3.9e−04
C-C motif chemokine 3 (CCL3)	0.11	3.6e−02	2.14	1.10–4.26	2.7e−02	0.33	7.1e−03	6.10	2.25–18.39	6.8e−04
C-C motif chemokine 4 (CCL4)	0.16	1.7e−02	1.86	1.13–3.12	1.7e−02	0.27	2.7e−02	2.60	1.36–5.30	5.7e−03
C-C motif chemokine 20 (CCL20)	0.33	7.1e−03	1.50	1.15–2.01	3.8e−03	0.39	3.3e−02	1.65	1.09–2.63	2.5e−02
C-C motif chemokine 25 (CCL25)	0.18	2.0e−02	2.03	1.28–3.26	3.0e−03	0.21	7.6e−02	2.30	1.20–4.64	1.5e−02
C-X-C motif chemokine 16 (CXCL16)	0.14	1.3e−04	6.59	2.37–19.40	4.2e−04	0.14	8.4e−03	8.06	1.66–45.79	1.3e−02
Chitinase-3-like protein 1 (CHI3L1)	0.25	1.7e−03	1.95	1.24–3.16	5.1e−03	0.33	2.3e−02	2.12	1.25–3.85	8.4e−03
CUB domain-containing protein 1 (CDCP1)	0.29	1.5e−05	4.08	2.09–8.36	6.6e−05	0.19	4.5e−02	4.25	1.56–13.11	7.6e−03
Fms-related tyrosine kinase 3 ligand (Flt3L)	0.24	2.7e−04	2.95	1.59–5.65	8.0e−04	0.14	8.4e−02	3.29	1.21–9.51	2.2e−02
Folate receptor alpha (FR-alpha)	0.19	2.1e−04	4.01	1.98–8.52	1.8e−04	0.16	2.1e−02	8.69	2.41–35.49	1.5e−03
Galectin-3 (Gal-3)	0.10	3.0e−02	2.79	1.20–6.68	1.9e−02	0.20	7.1e−03	6.33	1.95–23.46	3.5e−03
Growth/differentiation factor 15 (GDF-15)	0.34	1.2e−07	6.48	3.15–14.37	1.3e−06	0.25	6.9e−03	8.47	2.95–27.83	1.8e−04
Interleukin-10 (IL-10)	0.25	1.0e−02	2.26	1.39–3.98	2.4e−03	0.25	3.7e−02	2.47	1.16–5.64	2.5e−02
Interleukin-10 receptor subunit beta (IL-10RB)	0.14	1.5e−03	2.77	1.32–6.02	8.3e−03	0.19	8.4e−03	4.05	1.34–13.38	1.7e−02
Interleukin-12 (IL-12)	0.22	2.2e−02	1.73	1.18-2.57	5.5e−03	0.34	1.3e−02	2.14	1.18–4.03	1.5e−02
Interleukin-12 subunit beta (IL-12B)	0.22	4.9e−03	2.09	1.33–3.36	1.7e−03	0.31	1.6e−02	2.12	1.13–4.16	2.2e−02
Interleukin-17 receptor B (IL-17RB)	−0.19	1.2e−02	0.58	0.36–0.92	2.2e−02	-0.21	2.5e−02	0.39	0.16–0.89	3.1e−02
Kallikrein-6 (KLK6)	0.16	5.4e−05	6.34	2.81–15.14	1.6e−05	0.10	1.2e−01	3.80	1.25–12.66	2.3e−02
Matrilysin (MMP-7)	0.31	7.5e−09	6.28	3.15–13.30	5.4e−07	0.24	1.4e−03	7.04	2.48–22.59	5.0e−04
Placenta growth factor (PGF)	0.08	1.0e−02	5.60	1.72–19.12	4.9e−03	0.11	6.8e−02	4.35	1.12–18.42	3.8e−02
Pro-adrenomedullin (AM)	0.31	1.4e−07	5.30	2.68–11.02	3.6e−06	0.21	2.3e−02	2.64	1.14–6.46	2.7e−02
Procathepsin L (CTSL1)	0.13	1.5e−03	4.07	1.72–10.40	2.2e−03	0.23	4.7e−04	25.43	6.28–125.49	2.0e−05
Prostasin (PRSS8)	0.22	1.7e−06	5.57	2.51–13.06	4.2e−05	0.18	2.3e−02	6.21	2.00–21.16	2.2e−03
Protransforming growth factor alpha (TGF-alpha)	0.33	4.2e−05	2.21	1.44–3.50	4.7e−04	0.44	1.7e−04	3.25	1.67–6.76	8.7e−04
Renin (REN)	0.24	8.4e−04	2.60	1.60–4.39	2.0e−04	0.22	4.5e−02	2.87	1.36–6.39	7.2e−03
Tumor necrosis factor ligand superfamily member 13B (BAFF)	0.15	2.6e−03	2.53	1.30–5.06	7.2e−03	0.17	3.7e−03	6.39	1.51–30.24	1.5e−02
Tumor necrosis factor receptor superfamily member 10B (TRAIL-R2)	0.14	2.7e−04	6.61	2.35–19.99	5.3e−04	0.15	7.7e−03	6.84	1.64–32.45	1.1e−02
Tumor necrosis factor receptor superfamily member 1A (TNF-R1)	0.12	2.6e−04	4.88	1.78–14.09	2.6e−03	0.16	2.7e−03	13.09	2.69–73.81	2.2e−03
Tumor necrosis factor receptor superfamily member 1B (TNF-R2)	0.16	3.5e−04	3.37	1.59–7.45	2.0e−03	0.19	7.1e−03	4.41	1.44–14.71	1.2e−02
Tumor necrosis factor receptor superfamily member 6 (FAS)	0.13	1.3e−02	3.84	1.58–10.40	5.5e−03	0.11	7.6e−02	5.19	1.25–24.06	2.8e−02
Tumor necrosis factor receptor superfamily member 9 (TNFRSF9)	0.13	9.3e−03	2.37	1.22–4.79	1.3e−02	0.21	1.5e−02	3.94	1.49–11.61	8.5e−03
WAP four-disulfide core domain protein 2 (HE4)	0.21	4.4e−05	4.74	2.20–10.65	1.1e−04	0.18	7.7e−03	11.90	3.25–50.04	3.5e−04

*SBP-S/G* St. Göran Bipolar Project Stockholm/Gothenburg, *FDR* false discovery rate, *OR* odds ratio, *95% CI* 95% confidence interval.

^a^Two-sided *T*-test.

^b^Covariate-adjusted logistic regression.

To explore the prototypical form bipolar disorder, we repeated the above analyses including only cases with bipolar disorder type 1 and controls. In this restricted analysis, 64 proteins in SBP-S and 73 proteins in SBP-G were significantly (FDR < 0.05) associated with bipolar type 1, and 28 proteins were considered replicated from the logistic regression analyses (Supplementary Table 3).

### Sensitivity analyses

Somatic comorbidities were more common in cases than controls and may implicate pathways where case-control-associated proteins are involved. We, therefore, conducted a sensitivity case-control analysis excluding individuals with major somatic comorbidities (*n* = 45 in SBP-S and *n* = 11 in SBP-G). In this analysis, 62 and 51 proteins were significantly (FDR < 0.05) associated with bipolar disorder in SBP-S and SBP-G, respectively. Out of the 32 replicated proteins from the primary analysis, seven proteins failed to reach replicated statistical significance (*P* < 0.05) in covariate-adjusted logistic regression models: CCL3, CCL4, AR, TRAIL-R2, TNF-R2, AM, and PGF (Supplementary Table 4).

### Associations with psychiatric drugs

As controls were not exposed to psychotropic drugs, we were not able to adjust our results for this potential confounder. Instead, we estimated the variance in serum protein concentrations explained by psychiatric drugs (lithium, anticonvulsant mood stabilizers, antipsychotics, and antidepressants). In this analysis, we merged cases from the two cohorts (*n* = 224 + 100) and analyzed all included proteins. The strongest drug-protein association was observed for MMP-7, where lithium explained 20% of the total variance (*η*^2^ = 0.20, *P* < 3.5 × 10^−17^). Several other protein concentrations were associated with lithium use, *e.g*., KLK6 (η^2^ = 0.08, *P* = 1.4 × 10^−7^) and E-selectin (*η*^2^ = 0.08, *P* = 2.6 × 10^−7^), whereas TRANCE was associated with antipsychotic use (*η*^2^ = 0.08, *P* = 2.5 × 10^−7^) (Fig. 1b, Supplementary Table 5).

Given the strong association of MMP-7 and lithium use, we conducted post-hoc analyses (Supplementary fig. 2). First, we tested case-control associations per cohort stratified by lithium use. Whereas patients on lithium had higher concentration of MMP-7 than controls (*P* = 3.1 × 10^−16^/8.2 × 10^−10^ in SBP-S and SBP-G, respectively), patients without lithium did not differ from controls (*P* < 0.05, Fig. 1c). Further, the serum concentration of MMP-7 was positively associated with S-lithium (β (95% CI) = 0.62 (0.32–0.93), *P* = 1.0 × 10^−4^) and duration of lithium treatment in months (β (95% CI) = 0.002 (0.001–0.003), *P* = 2.7 × 10^−05^). In the subset of patients that had recently started lithium therapy (duration ≤ 1 month, *n* = 11), we also found a positive association of S-lithium and the serum concentration of MMP-7 (β (95% CI) = 1.81 (0.08–3.53), *P* = 0.04). Finally, we tested for association with eGFR and found no significant association for MMP-7 alone, but a negative interaction effect with the serum concentration of MMP-7 and use of lithium (*P* = 0.023, Supplementary Table 6).

### A biomarker classifier

**Fig. 2:**
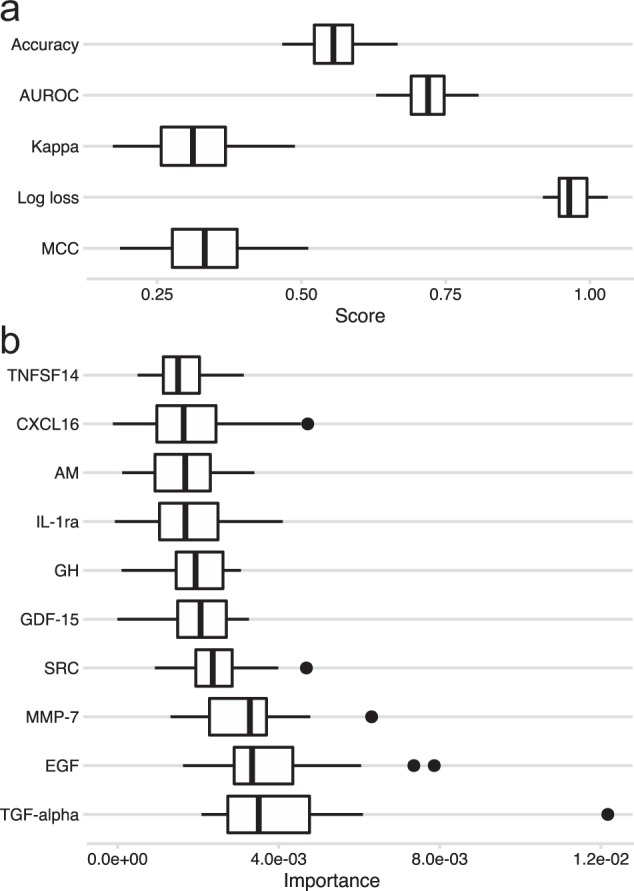
Overview of classifier performance. Boxplots showing (**a**) classification metrics (accuracy, area under receiver operating curve (AUROC), Cohen’s kappa, Matthew’s correlation coefficient (MCC), log loss), and (**b**) importance score for the ten most influential proteins across the 25 outer loops (5 folds × 5 repeats). CXCL16 C-X-C motif chemokine 16, IL-1ra Interleukin 1 receptor alpha, AM Pro-adrenomedullin, GH Somatotropin, SRC proto-oncogene tyrosine-protein kinase Src, GDF-15 growth/differentiation factor 15, MMP-7 matrilysin, EGF pro-epidermal growth factor, TGF-alpha protransforming growth factor alpha, TNFSF14 tumor necrosis factor ligand superfamily member 14.

To assess the diagnostic potential of our studied serum biomarkers, we developed a machine learning classifier for the bipolar subtypes and controls (Fig. 2). The average classification metrics across the three classes were: accuracy = 0.56, AUROC = 0.72, kappa = 0.32, MCC = 0.33, log loss = 0.97. Controls and bipolar type 1 had higher kappa statistics (0.41 and 0.29, respectively) than bipolar type 2 (0.09). TGF-alpha, Pro-epidermal growth factor (EGF), MMP-7, proto-oncogene tyrosine-protein kinase Src (SRC), and GDF-15 were the five most influential proteins. Finally, we retrained the model while excluding proteins suspected to be influenced by psychotropic drugs (listed in Fig. 1b) and obtained similar performance (accuracy = 0.54, AUROC = 0.71, kappa = 0.29, MCC = 0.31, log loss = 0.98). EGF, GDF-15, and CCL4 were ranked highest in this model (Supplementary Fig. 3). Concentrations of the VIP proteins across the subtypes are shown in Supplementary Fig. 4.

## Discussion

We conducted a large-scale serum proteomics study in two independent case-control cohorts (total *n* = 493) to identify novel diagnostic biomarkers for bipolar disorder. In our primary case-control analysis, we identified 32 proteins significantly associated with bipolar disorder where results replicated in both cohorts adjusted for relevant covariates. Although the study design did not allow us to control for the potential impact of psychotropic drugs, we estimated the influence of psychiatric drugs on protein concentrations (cases only) and found that some case-control associations may have been driven by specific drugs. The most notable drug-protein association was a higher concentration of MMP-7 in patients treated with lithium (replicated across cohorts). Further, the MMP-7 concentration was positively associated with S-lithium concentration and duration of lithium treatment, as well as negatively associated with eGFR in individuals treated with lithium. Finally, we estimated the diagnostic potential of the studied serum biomarkers in a machine learning model, where the average classification performance in unseen test data was fair to moderate.

Our primary analysis identified 32 biomarkers that were associated with bipolar disorder in both cohorts. Of these, 22 are novel with respect to bipolar disorder, while serum or plasma concentrations of AM [32], CCL3 [33–35], CCL4 [33, 36], CCL20 [37], CCL25 [38, 39], CHI3L1 [40, 41], GDF-15 [39], IL-10 [39], MMP-7 [37], and TNF-R1 [39] have previously been reported to be higher in patients with bipolar disorder than controls. In line with the inflammatory hypothesis of bipolar disorder [42], we report altered levels of both proinflammatory (IL-17RB, IL-12, IL-12B) and regulatory (IL-10) cytokines and receptors, as well as chemokines (CXCL16, CCL3, CCL4, CCL20, CCL25), whereof only CCL3 was suggestively associated with medication. Several prior studies have associated bipolar disorder with altered concentrations of IL-1 receptor antagonist [18, 35, 43, 44] and IL-6 [33–35], which in our results did not quite reach statistical significance in both cohorts when controlling for relevant covariates. With respect to central nervous system processes, KLK6—a serine protease that degrades for example amyloid precursor protein [45] and alpha-synuclein [46, 47]—was associated with lithium use, which is interesting as lithium has shown protective effects for dementia [48]. Most other identified biomarkers are widely expressed in several tissues and cell types and have general roles in cell biology, such as growth regulation (e.g., GDF-15, PGF, TGF-alpha, AR), and tissue remodeling (e.g., PRSS8, CTSL).

The serum concentration of MMP-7 was associated with lithium use, S-lithium concentration, duration of lithium treatment, and inversely associated with eGFR in an interaction with lithium. MMP-7 is an endopeptidase targeting a broad set of substrates (*e.g*., collagen, Fas ligand [49], E-cadherin [50]) and is transcriptionally regulated by the canonical Wnt/beta-catenin signaling pathway where lithium acts [51]. MMP-7 is involved in fibrotic development across tissues [52, 53], and specifically in kidney disorders where it has been suggested to mediate tubulointerstitial fibrosis [50, 54]. In the diseased kidney, MMP-7 is detected in tubular [50, 55] and cyst-lining epithelium [56], and can cause proteinuria by cleaving slit diaphragm proteins [57]. Lithium-induced chronic nephropathy is characterized by tubulointerstitial fibrosis out of proportion to the vascular or glomerular injury, microcystic dilated tubules, and (less specific) proteinuria [5, 58–61]. Both lithium [62] and MMP-7 have initial protective effects for acute kidney injury, supposedly by priming tubular epithelial cells for survival and regeneration [63]. Altered tubular cell turnover is also believed to induce the tubular microcyst formation seen with long-term lithium exposure [64]. Interestingly, serum and urinary levels of MMP-7 can predict progression across multiple kidney disease states [65–67] and reflect the renal fibrotic stage [54, 68]. Moreover, fibrotic development can be mitigated by both inhibition of MMP-7 activity [50, 69] and blockage of lithium reabsorption in tubules [70]. Taken together, our findings combined with previous literature implicate MMP-7 in the renal effects of lithium and future studies (i.e., animal models) are encouraged to further explore this hypothesis. No association with lithium use was found for other Wnt/beta-catenin interactors (dickkopf-related protein 1, axin-1).

We used machine learning to estimate the diagnostic potential of the studied serum biomarkers. The overall classification performance was fair to moderate and seemed to be better in controls and bipolar type 1 than type 2. This is interesting as type 1 is the prototypal bipolar manifestation and a more homogenous subgroup compared to type 2. Drug-associated proteins were influential but not critical to the classification performance as similar metrics were obtained in the model without those proteins. We acknowledge, however, the limited utility of the proposed classifier. A clinically viable diagnostic tool must show relevant performance in several stages of the disorder (*e.g*., in premorbid or drug-naïve cases) as well as across disorders, none of which are represented in our sample.

By using proximity extension assay targeted towards an explorative set of biomarker candidates in a well-powered and meticulously phenotyped sample, our study addresses several limitations that have hampered biomarker research in psychiatry [71]. Yet, there are several limitations to consider. First, blood sampling of cases preceded that of controls by 2–3 years in SBP-S. Long-term storage may impact protein concentrations, although this effect is typically seen in decades rather than years [72]. Moreover, SBP-S samples had a slight post-centrifugation delay to freezer (while keeping low temperature), but the collection procedure did not differ between cases and controls. For some proteins (e.g., EGF, SRC, CD69), we observed clear case-control differences in SBP-S that did not replicate in SBP-G. This difference was not driven by somatic comorbidity, but whether pre-analytical factors have impacted these results remains unclear. Second, we observed some clinical differences between the two cohorts that might impact replicability. Still, we identify 32 proteins qualifying a stringent definition of replication. Third, in the absence of a drug-naïve bipolar subgroup, this study was not designed to fully explore the impact of psychoactive drugs on serum protein levels. Moreover, cross-sectional studies may be biased by unmeasured exposures (e.g., lifestyle, diet) and our conclusions must thus be interpreted with the naturalistic design in consideration.

## Conclusion

We identified 32 proteins biomarkers associated with bipolar disorder that replicated in two independent case-control cohorts. Further, we identified an association between serum concentration of MMP-7 and lithium use. Future studies are encouraged to further explore the role of MMP-7 in lithium-induced chronic nephropathy, where MMP-7 could potentially serve as a predictive biomarker for early detection of chronic kidney injury.

## Supplementary information

Supplementary Material

Supplementary Tables
