# Supplementary material for: A serum proteomic study of two case-control cohorts identifies novel biomarkers for bipolar disorder
Source: Transl Psychiatry. 2022 Feb 8;12:55. doi: 10.1038/s41398-022-01819-y (PMC8826439; doi:10.1038/s41398-022-01819-y)
Supplement: Supplementary file 1 — Supplementary Material [file 41398_2022_1819_MOESM1_ESM.docx]

# Matrilysin (MMP-7) as a potential biomarker for lithium-induced nephropathy: a serum proteomic study of two bipolar disorder cohorts

*Supplementary* *material*

Index

p. 2 List of specific drugs compiled in the 4 drug categories

p. 3 Supplementary figure 1. Protein-protein interaction network for the 32 replicated proteins

p. 4 Supplementary figure 2. Matrilysin (MMP-7) in relation to lithium and eGFR

p. 5 Supplementary figure 3. Classification metrics and importance from the sensitivity machine learning classifier

p. 6 Supplementary figure 4. Violin plots of the most influential proteins

*Supplementary tables.xlsx* contains 1) a list of all tested proteins and which were included for analysis; 2-4) results from case-control analyses; 5) Results from drug-protein associations

# Supplementary list: drug categories

*Lithium* (Li): Lithium.

*Anticonvulsants* (AC): Lamotrigine, valproate, carbamazepine, topiramate, clonazepam, oxcarbazepine, pregabalin.

*Antipsychotics* (AP): Olanzapine, quetiapine, zuclopenthixol, ziprasidone, risperidone, clozapine, aripiprazole, haloperidol, perphenazine, flupentixol.
Not included: alimemazine, levomepromazine

*Antidepressants* (AD): Sertraline, venlafaxine, paroxetine, nortriptyline, moclobemide, mirtazapine, clomipramine, fluoxetine, escitalopram, duloxetine, citalopram, bupropion, amitriptyline, agomelatine.

# Supplementary figure 1


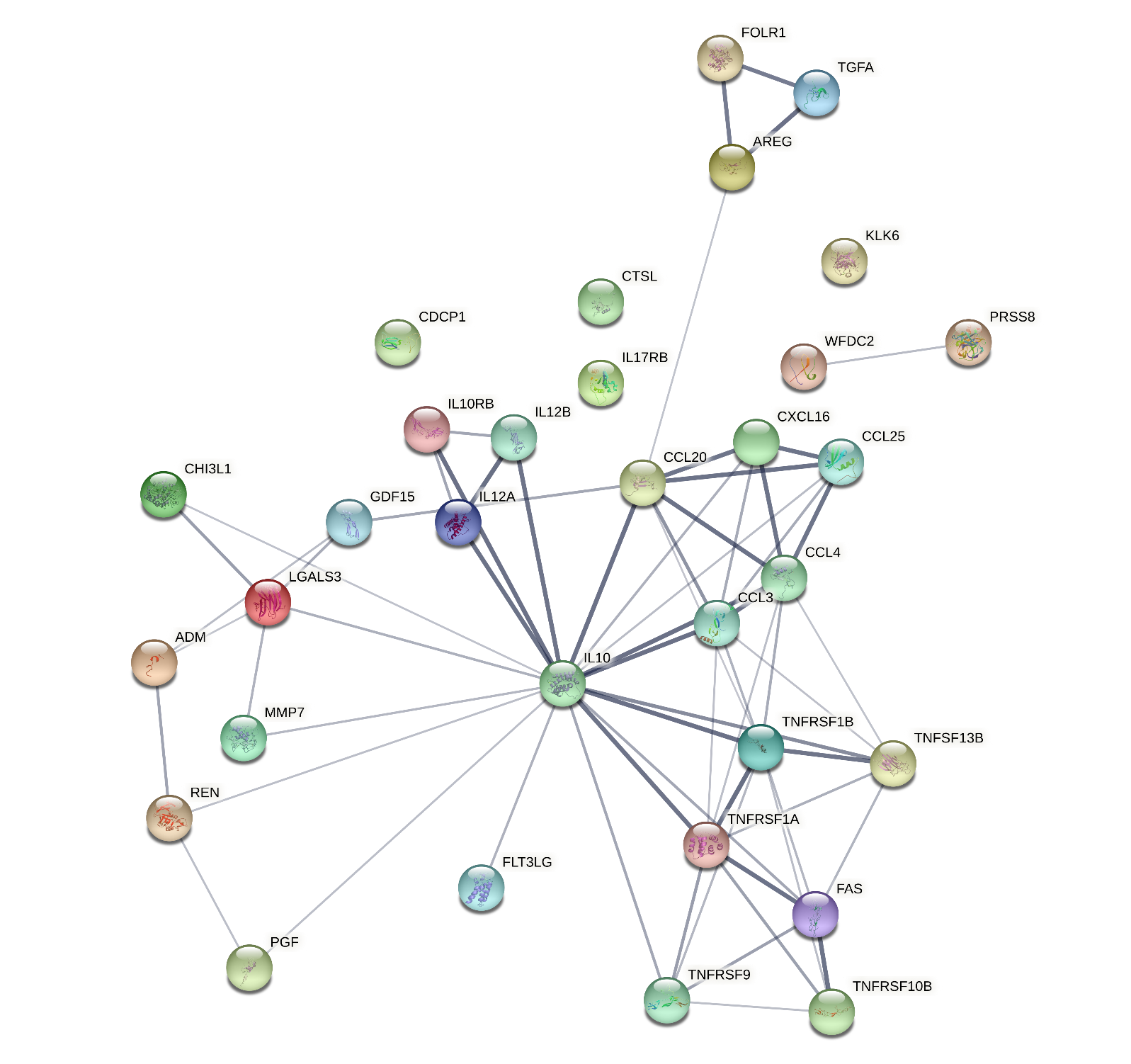


Supplementary Figure 1. Protein-protein interaction network of the 32 replicated proteins as provided by [www.string-db.org](http://www.string-db.org). Edge density is mapped to interaction score – thick lines represent more reliable predictions.

# Supplementary figure 2


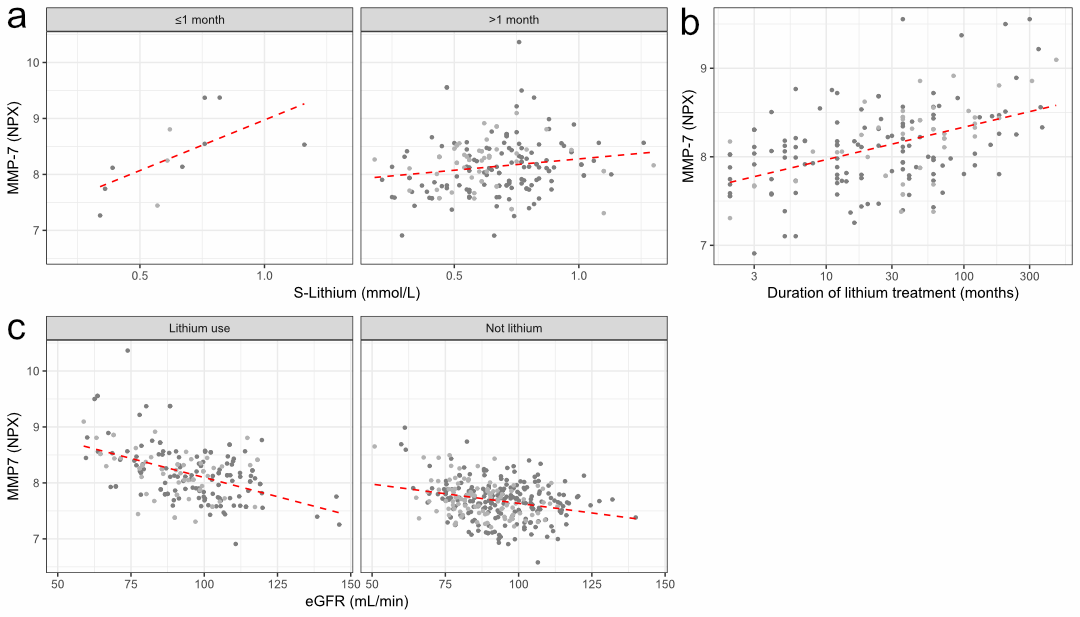


Supplementary figure 2. Serum concentration of matrilysin (MMP-7) related to a) serum concentration of lithium (S-lithium, mmol/L) in patients treated with lithium less than or equal to one month (left) and more than one month (right); b) duration of lithium treatment in months for individuals with >1 month treatment duration; c) estimated glomerular filtration rate (eGFR, mL/min) in cases with lithium (left) and cases and controls without lithium (right). Dashed red line indicates linear regression line.

# Supplementary figure 3


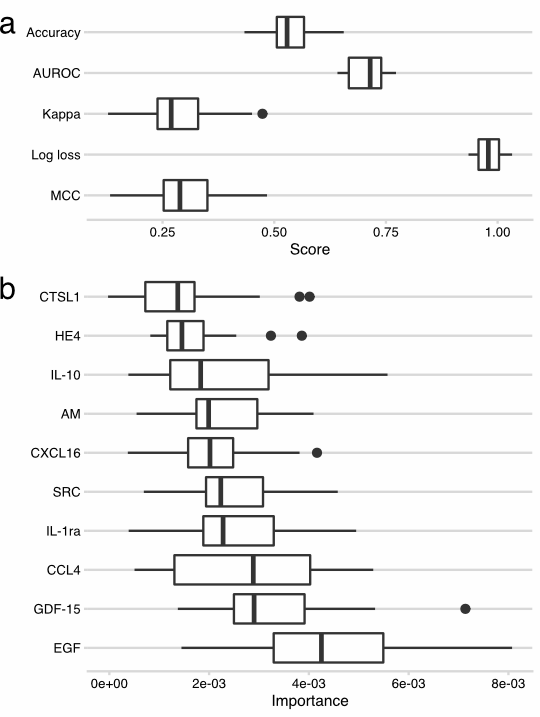


Supplementary Figure 3. Boxplots from the sensitivity classification model trained without drug-associated proteins. a) Classification metrics (accuracy, area under receiver operating curve (AUROC), Cohen’s kappa, Matthew’s correlation coefficient (MCC), log loss). b) Importance score for the ten most influential proteins across the 25 outer loops (5 folds x 5 repeats).

# Supplementary figure 4. Boxplots for VIP proteins


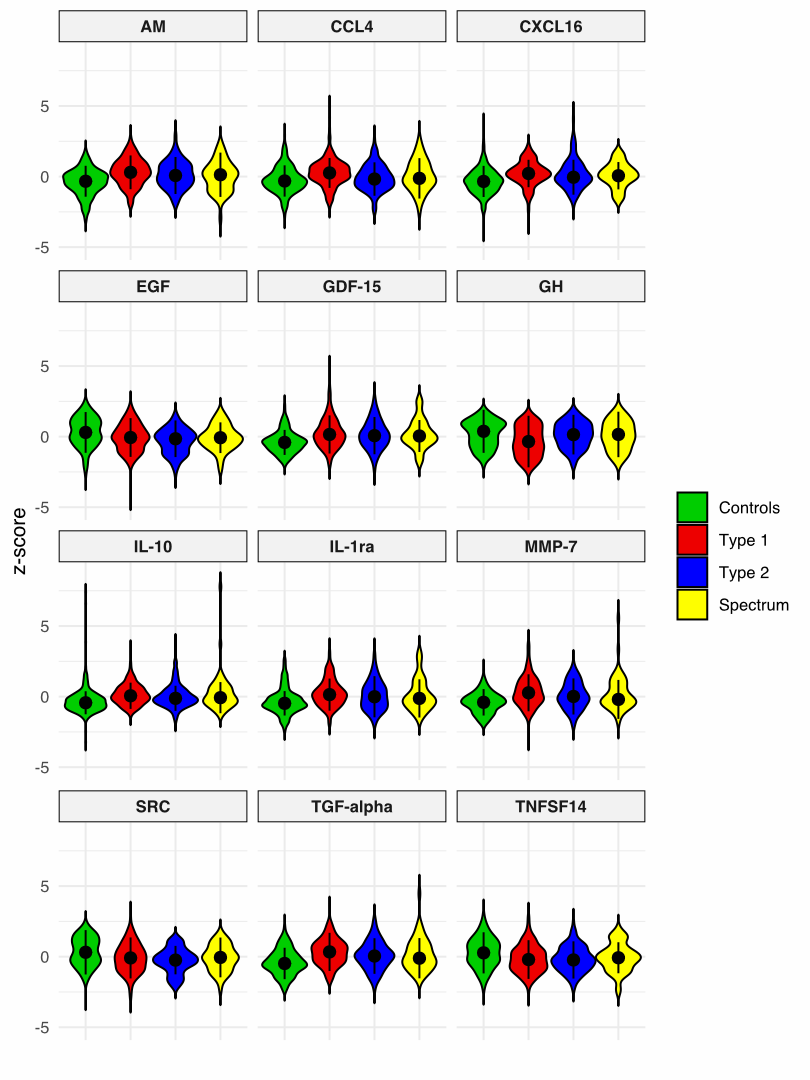


Supplementary figure 4. Showing boxplots from the top 12 most influential proteins in the machine learning classifier. NPX-values are standardized to z-scores (mean=0, standard deviation=1).
